# Varying Response of the Concentration and Yield of Soybean Seed Mineral Elements, Carbohydrates, Organic Acids, Amino Acids, Protein, and Oil to Phosphorus Starvation and CO_2_ Enrichment

**DOI:** 10.3389/fpls.2016.01967

**Published:** 2016-12-27

**Authors:** Shardendu K. Singh, Jinyoung Y. Barnaby, Vangimalla R. Reddy, Richard C. Sicher

**Affiliations:** ^1^Crop Systems and Global Change Laboratory, United States Department of Agriculture – Agricultural Research Service, BeltsvilleMD, USA; ^2^Wye Research and Education Center, University of Maryland, College Park, College ParkMD, USA

**Keywords:** contents, glycerate, myo-inositol, pinitol, phytic acid, proline, putrescine

## Abstract

A detailed investigation of the concentration (e.g., mg g^-1^ seed) and total yield (e.g., g plant^-1^) of seed mineral elements and metabolic profile under phosphorus (P) starvation at ambient (aCO_2_) and elevated carbon dioxide (eCO_2_) in soybean is limited. Soybean plants were grown in a controlled environment at either sufficient (0.50 mM P, control) or deficient (0.10 and 0.01 mM, P-stress) levels of P under aCO_2_ and eCO_2_ (400 and 800 μmol mol^-1^, respectively). Both the concentration and yield of 36 out of 38 seed components responded to P treatment and on average 25 and 11 components increased and decreased, respectively, in response to P starvation. Concentrations of carbohydrates (e.g., glucose, sugar alcohols), organic acids (e.g., succinate, glycerate) and amino acids increased while oil, and several minerals declined under P deficiency. However, the yield of the majority of seed components declined except several amino acids (e.g., phenylalanine, serine) under P deficiency. The concentration-based relationship between seed protein and oil was negative (*r*^2^ = 0.96), whereas yield-based relationship was positive (*r*^2^ = 0.99) across treatments. The CO_2_ treatment also altered the concentration of 28 out of 38 seed components, of which 23 showed decreasing (e.g., sucrose, glucose, citrate, aconitate, several minerals, and amino acids) while C, iron, Mn, glycerate, and oil showed increasing trends at eCO_2_. Despite a decreased concentration, yields of the majority of seed components were increased in response to eCO_2_, which was attributable to the increased seed production especially near sufficient P nutrition. The P × CO_2_ interactions for the concentration of amino acids and the yield of several components were due to the lack of their response to eCO_2_ under control or the severe P starvation, respectively. Thus, P deficiency primarily reduced the concentration of oil and mineral elements but enhanced a majority of other components. However, seed components yield consistently declined under P starvation except for several amino acids. The study highlighted a P nutritional-status dependent response of soybean seed components to eCO_2_ suggesting the requirement of an adequate P supply to obtain the beneficial effects of eCO_2_ on the overall yield of various seed components.

## Introduction

Crop growth and yield responses to either phosphorus (P) starvation or rising atmospheric carbon dioxide (CO_2_) concentration are well documented. However, responses of the nutritional aspects of crop yield particularly seed or grain to these factors or their interactions have rarely been explored. P deficiency reduces growth, yield, and seed quality of soybean (Ranjan et al., 1962; Israel and Rufty, 1988; Israel, 1991; Singh et al., 2014). However, elevated CO_2_ (eCO_2_) stimulates crop growth primarily through increased photosynthesis and leaf area (Cure et al., 1988; Prior et al., 1998; Ainsworth et al., 2002). Despite the opposite effects of these two factors on crops, studies that combine these factors have shown that eCO_2_ compensates, at least partially, for the adverse effects of P deficiency on plant growth and yield (Cure et al., 1988; Prior et al., 1998; Singh et al., 2013, 2014). Previous studies indicated alterations in the various seed components including minerals and metabolic profiles such as carbohydrates, organic acids, amino acids, protein, or oil, when plants were exposed to stress situations including P deficiency (Walker et al., 1985; Israel, 1991; Gifford et al., 2000; Prior et al., 2008; Rotundo and Westgate, 2009; Singh et al., 2014). Moreover, eCO_2_ had been shown to lessen the concentrations of minerals such as nitrogen, phosphorus, iron, and zinc, and protein in the seeds or edible parts of major crops (Högy et al., 2013; Loladze, 2014; Myers et al., 2014). The unwanted changes in the mineral elements or metabolic profiles due to environmental factors in seeds of the main food crops can have profound impacts on the human and animal nutrition.

Deficiency of a specific nutrient can also alter the dynamics of other elements in the plant organs by affecting the uptake and assimilation (Singh and Reddy, 2014; Singh et al., 2014; Gao and Ma, 2015). Previous reports showed that P deficiency increased both N concentrations and the N/P ratio of plant tissues (Singh et al., 2013, 2014). The tissue N/P ratio is a potential indicator of P limitation in crops (Koerselman and Meuleman, 1996; Singh et al., 2014). The soil nutrient status strongly affects the uptake and tissue concentration of mineral elements and metabolic profile (Prior et al., 2008). In general, at the early stage, plant leaves act as a sink for mineral nutrients and carbohydrates, while later they become nutrient sources for the new growth and seed development (Himelblau and Amasino, 2001). Hanway and Weber (1971) reported that approximately half the nutrients from vegetative tissues were mobilized to seeds during reproductive growth stages. However, the transport and metabolism of various seed constituents were affected by the tissue concentration of nutrients and external environmental factors. Despite the commonly observed lower tissue concentrations of mineral nutrients under CO_2_ enriched environments, evidence suggests greater nutrient demand by crop plants due to an overall increase in biomass accumulation (Conroy, 1992; Rogers et al., 1993; Singh et al., 2014). Thus, macronutrient deficits, such as low P concentrations, might exert substantial limitations on crop responses to eCO_2_. Factors such as dilution of tissue nutrients due to increased photosynthesis and plant size (biomass and yield), thicker leaves, and restricted uptake due to lower transpiration may contribute to the lower tissue nutrient concentrations at elevated CO_2_ (Gifford et al., 2000; Prior et al., 2008; Taub and Wang, 2008; Singh et al., 2014). However, the overall yield of nutrients on a per plant or area basis (also called as contents) are often greater under eCO_2_ because the total nutrient yield is a function of tissue concentration and total production of biomass or yield (Prior et al., 2008; Singh et al., 2015). Therefore, alterations of the plant growth, nutrient uptake and utilization under P deficiency and eCO_2_ are likely to affect the concentration and the total yield of seed components due to the changes in plant metabolic processes. Thus, it is important to recognize the effect of P deficiency on the constituents of grains in major row crops grown under ambient CO_2_ (aCO_2_) and eCO_2_. The response in the nutritional attributes of seeds to P deficiency will be useful to understand its nutritional impact and might offer an opportunity for quality improvement under P-stress.

Reports on the effect of P deficiency on the seed quality of grain crops, such as soybean, are extremely limited. Soybean [*Glycine max* (L.) Merr.] is one of the most important sources of protein and vegetable oil for both human and animal nutrition. Soybean seeds contain high levels of protein (≈ 42%) and oil (≈ 23%), making it one of the most versatile crops in the world (Dornbos and Mullen, 1992; Medic et al., 2014). Soybean seeds and soy products are also an important source of carbohydrates, amino acids, and mineral elements, which contribute to determining the overall nutritional value (Medic et al., 2014). In addition to genetic factors, environmental factors can considerably alter seed composition (Dornbos and Mullen, 1992; Rotundo and Westgate, 2009; Medic et al., 2014; Bellaloui et al., 2015). Moreover, the crop growth environments might have a varying impact between the concentration and total yield of a given seed constituents (Farhoomand and Peterson, 1968; Bauer et al., 1997). The concentration (e.g., mg g^-1^ seed) is an intensive property and reflect the percentage of a given constituent among other, whereas the total yield of a given constituent is an extensive property reflecting the total content (e.g., g plant^-1^). The concentration of a seed component is independent of the size of a sample, whereas the yield or content depends on the sample size or total production (Farhoomand and Peterson, 1968). Evidence suggests that the analysis of both the concentration and content of plant tissue components in a given species in response to an environment is important to fully characterize the nutritional quality and nutrient turnover (Bauer et al., 1997). Studies evaluating varying features between concentration and total yield of seed components of row crops in a given environment are extremely limited (Bruns, 2016).

Elemental P is an integral part of various metabolic processes, especially in energy transfer, that might affect the biosynthesis of seed constituents (Chiera et al., 2005). The majority of cropland is poor in P and rapidly depleting its resources, which is of major global concern (Cordell et al., 2009). Moreover, atmospheric CO_2_ concentration is expected to double from the current level of approximately 400 μmol mol^-1^ by the end of this century (IPCC, 2013). The seed filling processes of soybean depend on the remobilization of resources such as carbohydrates, minerals, and amino acids from plant organs, and plant photosynthesis capacity. All of these processes are influenced by P nutrition and eCO_2_, which in combination might affect seed quality traits in soybean (Israel, 1991; Conroy, 1992; Högy et al., 2013; Loladze, 2014; Myers et al., 2014; Gao and Ma, 2015). In the previous reports as a part of this study, we found alteration in the tissue N concentration and lower photosynthesis of soybean under P deficiency (Singh et al., 2014; Singh and Reddy, 2016), however, eCO_2_ stimulated growth, and seed yield that might have been influenced by the increased carbohydrate supply during the seed filling processes. Most of the prior studies on seed components often focused on the specific constituents, such as mineral elements or carbohydrates while other on the protein and oil or fatty acids. Also, the majority of these prior studies were conducted under natural environments and were complicated by the occurrence of heat and drought stresses (Ranjan et al., 1962; Prior et al., 2008; Högy et al., 2013; Krueger et al., 2013; Myers et al., 2014; Bellaloui et al., 2015; Gao and Ma, 2015). A comprehensive analysis of soybean seed quality under P deficiency and eCO_2_ is lacking. The objectives of this study were to determine the combined effect of P nutrition and growth CO_2_ on the concentrations and the yield of soybean seed mineral elements and metabolites. We hypothesize that (a) P deficiency will alter the concentrations but decrease the yield of seed components and (b) eCO_2_ will reduce the concentration of seed components but increase the yield, which might depend on the P nutritional-status of soybean.

## Materials and Methods

### Experiment Conditions

The experiment was conducted at USDA-ARS facility in Beltsville, MD, USA using six controlled environment growth chambers (EGC Corp., Chagrin Falls, OH, USA) in 2012. The experiment was repeated once over time. Soybean [*G. max* (L.) Merr., cv. Spencer] seeds were planted in pots (volume 7.6-L, one plant per pot) filled with washed sand in each chamber. Plants were watered with full-strength Hoagland’s nutrient solution (Hewitt, 1952) from emergence to 12 days after planting (DAP). After that the treatments were initiated in a combination of two levels of CO_2_ 400 μmol mol^-1^ (ambient, aCO_2_) and 800 μmol mol^-1^ (elevated eCO_2_) and three levels of P treatments 0.5 mM P (control), 0.10 and 0.01 mM P (P-stress) in the modified Hoagland’s nutrient solution. The nutrient solution was applied to flushed (300–350 mL) four – six times during the day. Pots were rotated periodically within each chamber to minimize the effects of within chamber heterogeneity. The CO_2_ treatments were also rotated between the repetitions of the experiment for each P treatment to minimize the potential chamber effect across CO_2_. Seven plants (one plant/pot) per chamber were maintained from 45 days after planting (DAP) to the maturity. A 28/22°C day/night (12 h/12 h) air temperature was maintained within ±0.15°C in the growth chambers during the experiment. The photosynthetically active radiation of 900 ± 15 μmol m^-2^ s^-1^ was maintained in the day within each chamber using controllable ballasts (Osram Sylvania, Wilmington, MA, USA). Injection of either CO_2_ or CO_2_-free air was determined using a TC-2 controller that monitored CO_2_ every 3 s measured from an absolute infrared gas analyzer (WMA-4PP-systems, Haverhill, MA, USA). More details of the experiment have been reported previously (Singh et al., 2014). Plants were harvested at maturity 110 DAP. Each plant for a given treatment was separately harvested and divided into plants parts. The total seed production from each plant was determined after drying the seed at 35°C in forced-ventilation air for 10 days and stored at 10°C for further analysis.

### Measurement of Seed Mineral Nutrients

The seeds from all plants in each treatment were ground separately using a Wiley Mill (Wiley^®^ Mill, Thomas Scientific, Swedesboro, NJ, USA) to pass through a 1 mm screen. The concentrations of seed carbon (C) and nitrogen (N) were determined by combustion of the ground seed material using a CHN-2000 (Carbon Hydrogen Nitrogen-2000: LECO Corporation, St. Joseph, MI, USA). The concentrations of seed mineral nutrients phosphorus (P), potassium (K), calcium (Ca), magnesium (Mg), sulfur (S), zinc (Zn), copper (Cu), boron (B), iron (Fe), and manganese (Mn), were determined in the ground seed-materials at the Agriculture Diagnostic Laboratory, University of Arkansas, Fayetteville, AR, USA, using a standard procedure (Plank, 1992). In brief, 0.25 g ground seed tissue was digested at 110–120°C for approximately 2 h using concentrated HNO_3_ and hydrogen peroxide on an Al digestion block in calibrated 50 ml tubes. Then the samples are brought to 25 ml total volume with deionized water. The digestates were analyzed by Spectro ARCOS EOP-Inductively Coupled Plasma (ICP) Spectrophotometer (Spectro Analytical Instruments, Mahwah, NJ, USA). The sodium (Na) concentration in soybean seeds could not be detected at a detection limit of 1.0 mg kg^-1^.

### Measurement of Seed Protein and Oil

The concentrations of seed protein and oil were determined non-destructively using near infrared (NIR) spectroscopy in a 25 g sample of randomly chosen mature soybean seeds from each plant of a given treatment. The NIR analyses were performed by the National Center for Agricultural Utilization Research (NCAUR), USDA-ARS, Peoria, IL, USA. In addition, protein concentration from the seed N concentration was also estimated using conversion factor 5.52 according to Mosse (1990).

### Measurement of Seed Metabolites

To determine metabolites approximately 30 mg of the ground seed material was suspended in 1.4 ml of 70% ice-cold methanol containing a final concentration of 2.5 mmol L^-1^ α-aminobutyric acid and 2 mg ml^-1^ ribitol as internal standards. The suspended samples were vortexed vigorously, allowed to stand at room temperature for 30 min and then centrifuged as described previously (Sicher, 2008). The pellets were extracted a second time with 1.4 ml of the above solvent, incubated in shaker bath for 15 min at 45°C, kept at room temperature for 30 min and centrifuged as above. The supernatants were combined in a 15 ml Falcon tube and stored at -20°C until use.

Soluble carbohydrates (sucrose, glucose, fructose, ribose, maltose, and pinitol) and organic acids (citrate, aconitate, succinate, shikimate, and glycerate) were measured by gas chromatography (HP/Agilent 6890A, Agilent Technologies, Wilmington, DE, USA) coupled to mass spectrometry. A 10 μl aliquot of each extract was transferred to a 1 ml reactivial and dried overnight in a desiccator under vacuum. The dried samples were dissolved in 100 μl of pyridine containing 20 mg ml^-1^ methoxyamine and the vials were incubated in a shaker bath for 90 min at 30°C. Subsequently, 100 μl of *N*-methyl-*N*-(trimethylsilyl) fluoroacetamide (MSTFA) was added to each vial, which was subsequently incubated in shaker bath for 30 min at 37°C. The derivatized samples were transferred to autosampler vials and crimp sealed prior to the analysis. Standard curves were prepared with four point curves using mixtures of known chemical standards.

Soluble amino acids [essential amino acids: valine (Val), phenylalanine (Phe), leucine (Leu), isoleucine (Ile); non-essential amino acids: glutamine (Gln), glycine (Gly), alanine (Ala), serine (Ser), proline (Pro), putrescine (Put, a ployamine produced by the breakdown of amino acids)] were also measured by gas chromatography-mass spectrometry as described by Wittmann et al. (2002). A 10–50 μl aliquot of each tissue extract was dried *in vacuo*, dissolved in 50 μl of dimethylformamide containing 0.1% pyridine and this was followed by 50 μl *N*-methyl-*N*-t-butyldimethylsilyl-trifluoroacetamide (MBDSTFA). The latter compound contained 1% TBDMCS (tert-butyldimethylchlorosilane) as a catalyst. Samples were sealed in crimp topped autosampler vials, vortexed gently and then heated for 30 min at 70°C in an Al heating block to complete the derivation process.

### Data Analysis

The yield of seed components (mineral elements, protein and oil, carbohydrates, organic acids, and amino acids) were obtained by multiplying their concentrations (e.g., g^-1^ dry weight, DW) with the total seed production (e.g., g plant^-1^; Singh et al., 2015). To test the effects of treatments and their interaction, the analysis of variance (ANOVA) was performed at α = 0.05 using the PROC MIXED procedure of SAS (SAS Enterprise Guide, 6.1, SAS Institute Inc., Cary, NC, USA). The P and CO_2_ treatments and their interactions were considered as fixed effects, and the repetition of the experiment was a random effect. The total number of the data point for each variable consisted of 14 individual plants, seven plants from each repetition of the experiment. The PROC REG procedure of SAS was used for regression analysis to calculate the coefficients as slope, intercept, the coefficient of determination (*r*^2^), and the level of significance (*P*-value).

## Results

### Majority of Minerals Concentration and Yield Tended to Decrease under P Starvation But eCO_2_ Increased C, Fe, and Mn Concentrations and Mineral Yields

**FIGURE 1:**
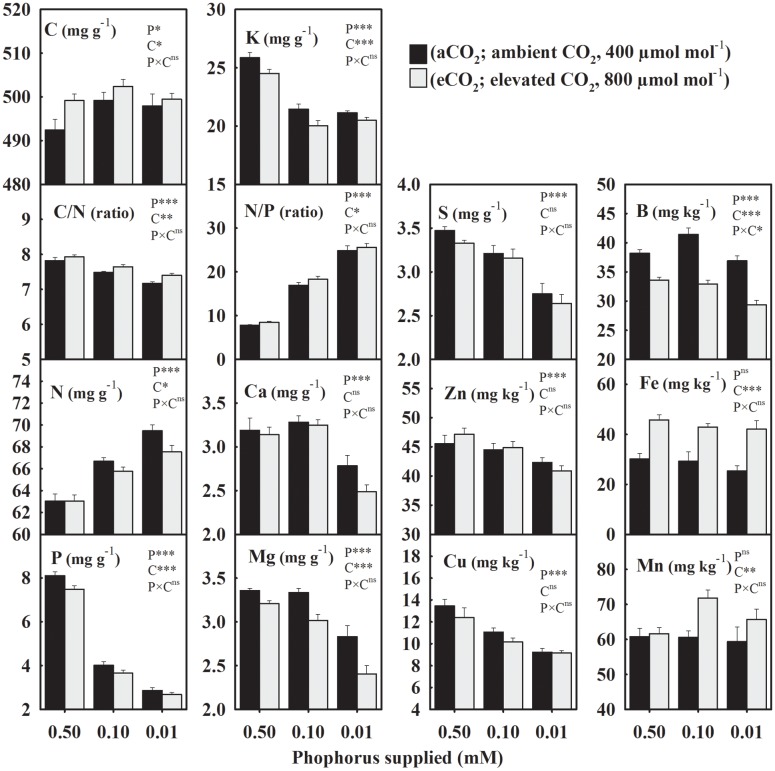
**The response of soybean mature seed mineral concentrations to three phosphorus (P) treatments supplied under ambient (aCO_2_, 400 μmol mol^-1^, black bars) and elevated (eCO_2_, 800 μmol mol^-1^, gray bars) CO_2_ levels.** C, carbon; N, nitrogen, C/N ratio; P, phosphorus; K, potassium; N/P ratio; Ca, calcium; Mg, magnesium; S, sulfur; Zn, zinc; Cu, copper; B, boron; Fe, iron; Mn, manganese. Bars represent mean ± SE (*n* = 14). The analysis of variance for the effect of treatments (phosphorus, P and CO_2_, C) and their interaction (P × C) is also shown by the significance levels, where ^∗^*P* ≤ 0.05, *^∗∗^P* ≤ 0.01, ^∗∗∗^*P* ≤ 0.001, and ^ns^*P* > 0.05, respectively.

The seed concentration of C, also considered as the non-mineral nutrient, increased from an average value of 495–500 mg g^-1^ under P deficiency (combined low P treatments, 0.10 + 0.01 mM P) versus control when averaged across CO_2_ levels (**Figure 1**). However, C/N decreased ≈ 5% under P-stress (**Figure 1**). Among the primary macronutrients, N increased 4.2–10.2%, but P and K concentrations decreased 51.2–64.6% and 16.4–18.3%, respectively, across growth CO_2_ (**Figure 1**). A substantial increase (116–218%) in the seed N/P ratio was observed under P deficiency. The concentrations of secondary macronutrients (Ca, Mg, and S) declined up to 20%, especially at the lowest P treatment. The average micronutrient concentrations of Zn and Cu decreased 3.6–18% at 0.10 mM P and 10.2–29% at 0.01 mM P treatments. Levels of B also declined ≈ 12.5% particularly in the lowest P treatment under eCO_2_. The Fe and Mn concentrations were not significantly affected by P treatment. Except, Ca, S, Zn, and Cu the concentration of other mineral elements were significantly affected by CO_2_ treatments (**Figure 1**). Averaged across P treatments, N, P, K, and Mg concentration decreased 1.4–9.4%, and B decreased by 17.7% when comparing eCO_2_ versus aCO_2_ (**Figure 1**). Seed C/N and N/P ratios increased 2.2–5.7% at eCO_2_ when averaged across P treatments. The C, Mn, and Fe concentrations were enhanced by eCO_2_ with the greatest increase observed for Fe (46–65%) across P treatments (**Figure 1**).

**FIGURE 2:**
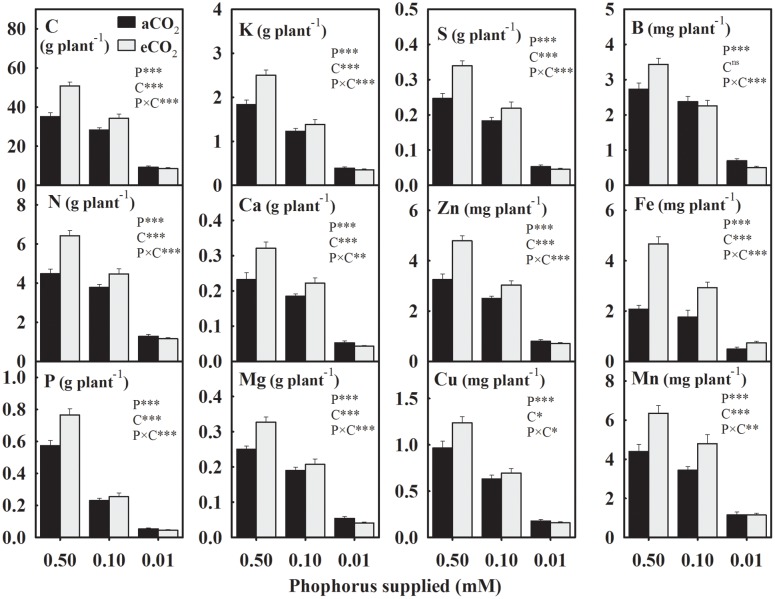
**The response of soybean mature seed mineral yield to three phosphorus (P) treatments supplied under ambient (aCO_2_, 400 μmol mol^-1^, black bars) and elevated (eCO2, 800 μmol mol^-1^, gray bars) CO_2_ levels.** Other details are as in **Figure 1**.

In general, the yield of mineral elements significantly declined with P supply exhibiting relatively greater decreases at 0.01 mM P or under eCO_2_ (**Figure 2**). For the aCO_2_, these mineral elements declined over 43% with the maximum decrease in the seed P yield by 75.2% under P deficiency (**Figure 2**). However, for the eCO_2_, these elements declined over 53.2% with the maximum decrease in the P yield by 80.4% under P-stress. In seeds obtained under eCO_2,_ the yield of mineral elements increased except at the lowest P treatment leading to the significant P × CO_2_ interactions (**Figure 2**). Averaged between two higher P treatments (0.50 and 0.10 mM P), the yield of most of the mineral elements increased between 18.8 and 41.7% at eCO_2_ versus aCO_2_ (**Figure 2**). The yield of Fe showed the greatest increase at the eCO_2_ by 95.8%, whereas the B yield was not affected by the CO_2_ treatment.

### Concentrations of Several Carbohydrates Tended to Increase But Yields Decreased by P Starvations. However, eCO_2_ Appeared to Show an Opposite Trend

**FIGURE 3:**
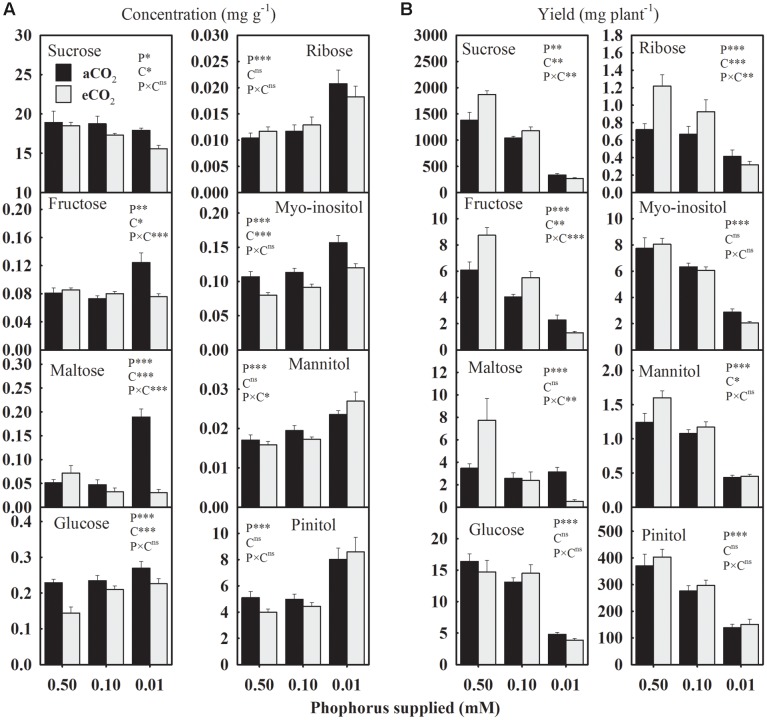
**The response of soybean mature seed carbohydrates** **(A)** concentrations (mg g^-1^) and **(B)** yield (mg plant^-1^) to three phosphorus treatments supplied under ambient (aCO_2_, 400 μmol mol^-1^, black bars) and elevated (eCO_2_, 800 μmol mol^-1^, gray bars) CO_2_ levels. Other details are as in **Figure 1**.

The P treatment significantly affected the concentration and the yield of seed carbohydrates (**Figure 3**). Averaged across CO_2_ levels, seed glucose, ribose, and sugar alcohols (myo-inositol, mannitol, and pinitol) concentrations consistently increased 28.9–44.0% under P-stress versus control (**Figure 3A**). The fructose and maltose concentrations showed an inconsistent response to P deficiency under aCO_2_ but tended to be lower at eCO_2_. Compared to the control, sucrose concentration declined up to 15.8% under P deficiency with the greater decreases observed at the lowest P treatment under eCO_2_. The eCO_2_ decreased (7.7–13%) sucrose concentration under P-stress, but not under the control (**Figure 3A**). Seed glucose and myo-inositol concentrations consistently declined 10.6–37.0% and 19.2–25.3%, respectively, at eCO_2_ versus aCO_2_ across P treatments. However, the changes in fructose and maltose concentrations between aCO_2_ and eCO_2_ varied depending on the P treatments.

In general, the yield of carbohydrates declined 11.3–55.9% at 0.10 mM P and 62.4–81.5% at 0.01 mM P versus control treatment when averaged across CO_2_ levels (**Figure 3B**). The eCO_2_ increased the yield of sucrose, fructose, ribose, and mannitol, especially at higher P (0.50 and 0.10 mM) supply, leading to P × CO_2_ interactions (**Figure 3B**). The yields of glucose, myo-inositol, maltose, and pinitol were not affected by CO_2_ treatments (**Figure 3B**).

### Organic Acids Concentration and Yields Tended to Increase and Decrease under P Starvation, Respectively. However, eCO_2_ Often Showed an Opposite Response Pattern

**FIGURE 4:**
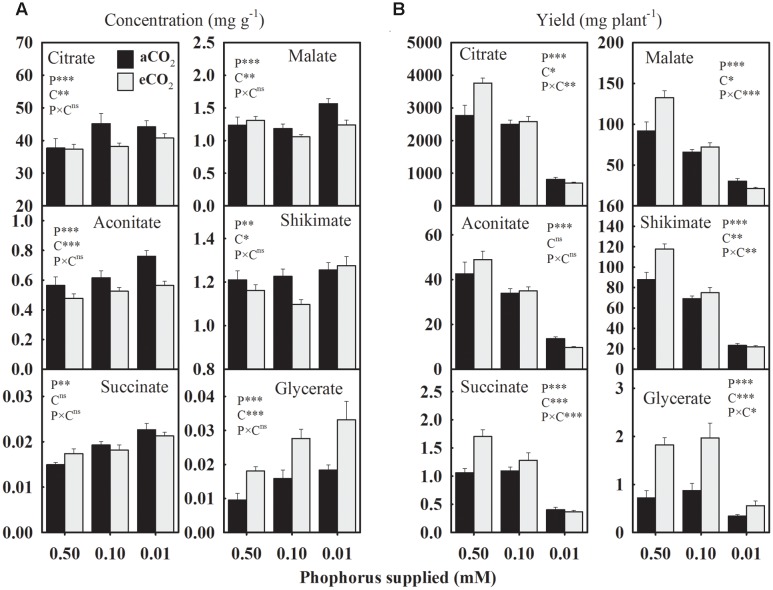
**The response of soybean mature seed organic acids** **(A)** concentrations (mg g^-1^) and **(B)** yield (mg plant^-1^) to three phosphorus treatments supplied under ambient (aCO_2_, 400 μmol mol^-1^, black bars) and elevated (eCO_2_, 800 μmol mol^-1^, gray bars) CO_2_ levels. Other details are as in **Figure 1**.

Due to P starvation, the seed concentrations of citrate, aconitate, succinate, and glycerate, increased by 12.1, 18.3, 25.8, and 72% compared to the control, respectively, when averaged across CO_2_ levels (**Figure 4A**). However, malate and shikimate concentrations showed a distinct response and tended to decrease at 0.10 mM P, but increase at the 0.01 mM P treatment. The eCO_2_ increased the glycerate concentration by 74.8–80% across P treatments but tended to decrease the concentrations of other organic acids, particularly under P deficiency (**Figure 4A**).

In general, the average yield of organic acids declined 14.0–38.5% (except glycerate) at 0.10 mM P and 64.9–78.0% at 0.01 mM P treatment (**Figure 4B**). The eCO_2_-mediated enhancements in the organic acids yields were dependent on the P treatment except for glycerate, which consistently increased 64–152% across P treatments (**Figure 4B**). However, citrate, succinate, malate, and shikimate yields showed a tendency to either increase or decrease in response to eCO_2_ at 0.50 mM and 0.01 P treatments, respectively.

### Protein and Oil Yields Declined But Concentration of Protein Increased under P Starvation. The eCO_2_ Tended to Increase the Concentration and Yield of Seed Oil

**FIGURE 5:**
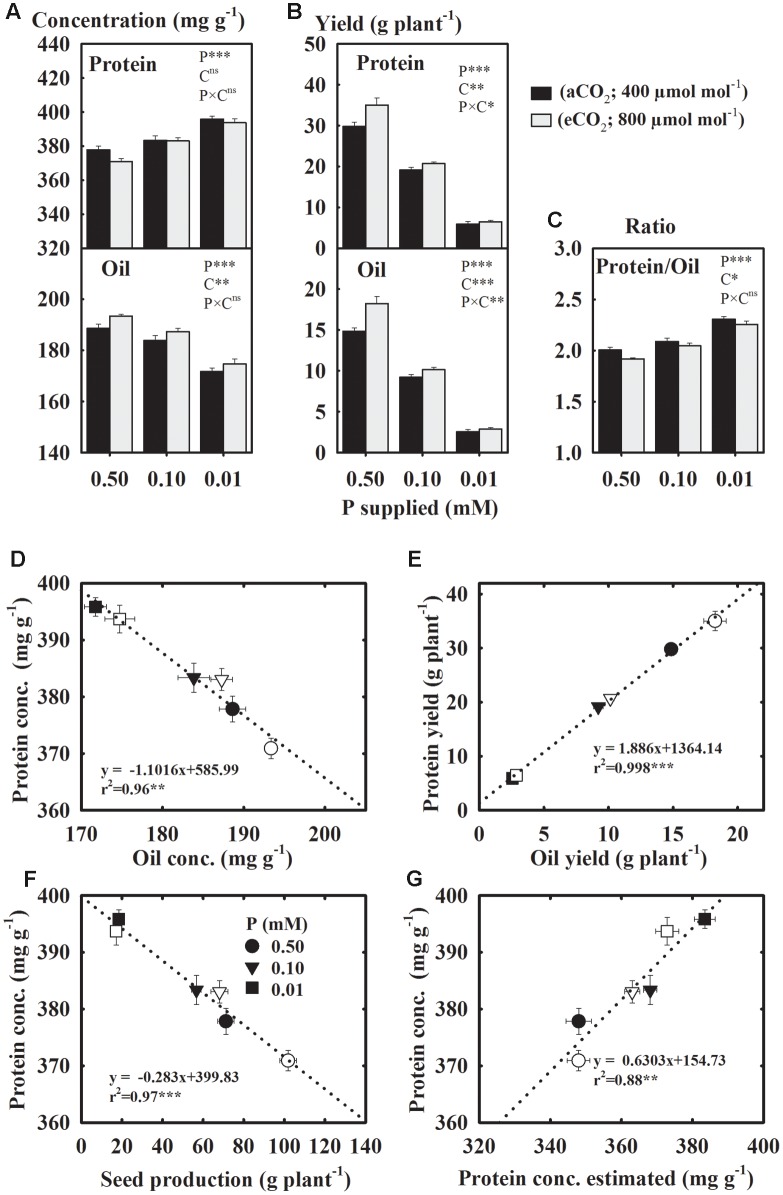
**Soybean mature seed concentration** **(A)** and yield **(B)** of protein and oil; **(C)** their ratio; the relationships between **(D)** protein and oil concentrations; **(E)** protein and oil yields, **(F)** total seed production and protein concentration, and **(G)** protein estimated using N concentration and measured protein. Soybean was grown under three levels of phosphorus (P) nutritions at ambient (aCO_2_, 400 μmol mol^-1^, black bars/filled symbols) and elevated (eCO_2_, 800 μmol mol^-1^, gray bars/unfilled symbols) CO_2_ levels. Bars/symbols represent mean ± SE (*n* = 14). The significance levels of analysis of variance **(A–C)** for the effects of treatments (phosphorus, P and CO_2_, C) and their interaction (P × C) and regression analysis **(D–G)** is also shown, ^∗^*P* ≤ 0.05, *^∗∗^P* ≤ 0.01, ^∗∗∗^*P* ≤ 0.001, and ^ns^*P* > 0.05, respectively. Error bars smaller than the symbols are not visible.

Compared to the control, seed protein concentration increased 2.4–5.4%, but oil concentration declined 2.8–9.3% under P starvation across CO_2_ (**Figure 5A**). The eCO_2_ enhanced the oil concentration but did not affect the concentration of protein. The yields of protein and oil declined 37.2–40.8% at 0.10 mM P and 78.2–82.1% at 0.01 mM P treatments as compared to the control (**Figure 5B**). However, the Protein/Oil ratio increased 5.4 and 10.3% at 0.10 and 0.01 mM P treatments, respectively (**Figure 5C**). The eCO_2_ stimulated the yields of both protein and oil (**Figure 5B**). There was an inverse linear (*r*^2^ = 0.96) relationship between the concentrations of protein and oil (**Figure 5D**), but their yields exhibited a positive relationship (*r*^2^ = 0.99) (**Figure 5E**). The protein concentration and total seed production also showed an inverse relationship (*r*^2^ = 0.97) (**Figure 5F**). There was a good agreement (*r*^2^ = 0.88) between the measured and estimated protein concentration from seed N concentration using the conversion factor 5.52 for soybean seeds (**Figure 5G**).

### Amino Acids Concentrations were Enhanced and Declined under P Starvation and eCO_2_, Respectively. However, Their Yields Varied Across the Treatments

**FIGURE 6:**
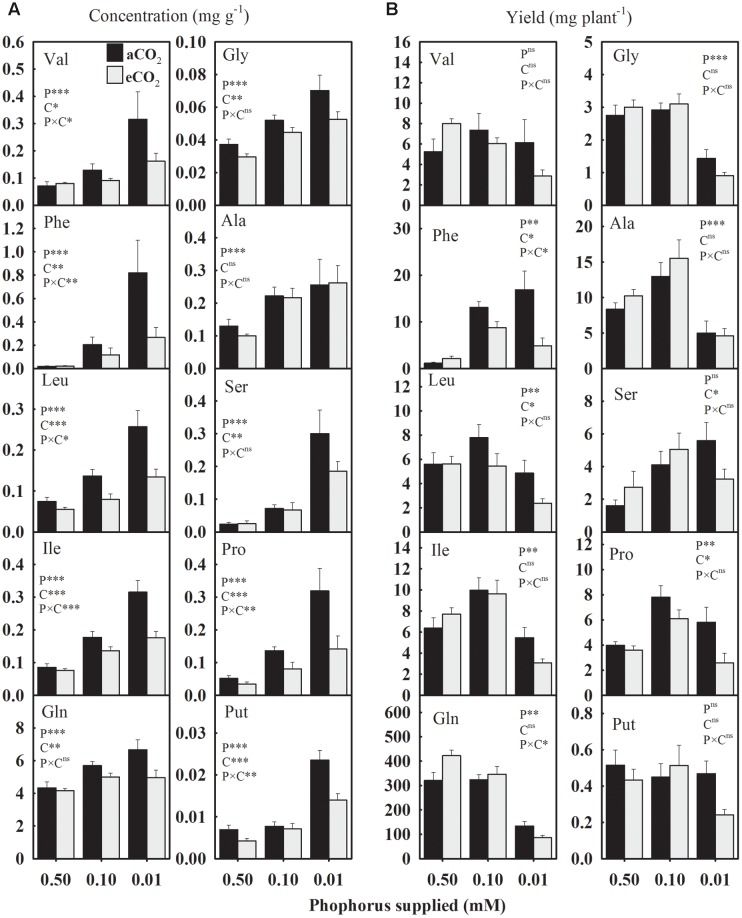
**The response of soybean mature seed amino acids** **(A)** concentrations (mg g^-1^) and **(B)** yield (mg plant^-1^) to three phosphorus treatments supplied under ambient (aCO_2_, 400 μmol mol^-1^, black bars) and elevated (eCO_2_, 800 μmol mol^-1^, gray bars) CO_2_ levels. Essential amino acids: Val, valine; Phe, phenylalanine; Leu, leucine; Ile, isoleucine. Non-essential amino acids: Gln, glutamine; Gly, glycine; Ala, alanine, Ser, serine; Pro, proline; and Put, Putrescine (a polyamine). Other details are as in **Figure 1**.

In general, seed amino acids concentrations were enhanced under P starvation compared to the control (**Figure 6A**). The concentration of essential amino acids (Val, Phe, Leu, and Ile) exhibited relatively a greater magnitude of increases at the lowest P treatment under aCO_2_ versus eCO_2_ (**Figure 6A**). Under aCO_2_, for example, Val concentration was enhanced ≈ 82.5 and 350% at 0.10 and 0.01 mM P, respectively. However, under eCO_2_, Val was increased 14 and 102% at 0.10 and 0.01 mM P, respectively. In response to P deficiency, the concentration of aromatic amino acid Phe showed the greatest increase of over 2700 and 800% (equivalent to 28- and 9-folds) at aCO_2_ and eCO_2_, respectively, compared to the control. The Gln was the most abundant accounting over 80% of the total measured amino acids. Averaged across CO_2_ levels, Gln and Gly concentrations were enhanced 25.8–44.5% and 36.9–83.7% at 0.10 and 0.01 mM P, respectively, as compared to the control. Such enhancements in Ala and Ser concentration under P-stress were approximately 91–182% and 126–892% at 0.10 and 0.01 mM P treatments, respectively. Averaged across CO_2_ levels, Put and Pro concentrations were also enhanced 33–151% and 236–435% at 0.10 and 0.01 mM P, respectively. The eCO_2_ substantially decreased the concentration of most of the amino acids under P starvation, but not under control P treatment leading to P × CO_2_ interactions. This eCO_2_-mediated decline in the concentration of amino acids, except Ala, ranged between 19.4 and 62.5% under P-stress (**Figure 6A**).

The yield of amino acids was significantly affected by P treatments except for Val and Put (**Figure 6**). Averaged across CO_2_ levels, Phe and Ser yields were enhanced ≈ 557 and 93%, respectively, under P deficiency as compared to the control (**Figure 6B**). However, Leu and Ile yields exhibited a distinct pattern and tended to increase at 0.10 mM P then declined at 0.01 mM P treatment. Compared to the control, the lowest P treatments decreased the yields of Gln and Gly 60–70% when averaged across CO_2_ levels. Compared to the control, Ala was enhanced ≈53% by 0.10 mM P but reduced ≈ 48% under 0.01 mM P treatment. The overall Pro yield also increased under P-stress except at the lowest P treatments under eCO_2_. The effect of CO_2_ was significant for Phe, Leu, and Pro leading to 36–54% decline in their yields, especially under P deficiency.

## Discussion

This study revealed the combined effect of P starvation and eCO_2_ on the concentration and yields of soybean seed components under well-watered conditions. The concentration of seed components varied in their sensitivity to P nutrition and growing environments (Ranjan et al., 1962; Israel, 1991; Rotundo and Westgate, 2009; Högy et al., 2013; Loladze, 2014; Medic et al., 2014; Bellaloui et al., 2015). The concentration of amino acids, in particular, showed P × CO_2_ interactions, which might be attributable to the lack of their response to eCO_2_ under control P treatment. Whereas, yields for majority of components exhibited P × CO_2_ interaction due to their minimal response at eCO_2_ under the lowest P treatment. The decline in seed and leaf tissues P concentration under P deficiency was reported previously by Singh et al. (2014) and was comparable to other observation in the field (Walker et al., 1985). Remarkably, several mineral elements declined due to P starvation, even though they were not limiting in the rooting media. However, the exceptions to this rule were N, which increased, and Fe and Mn, which were unaffected by P deficiency. Similarly, P deficiency did not affect canola seed Mn concentration when averaged across N-fertilization treatments (Gao and Ma, 2015). The observed high N concentration in P-stress seeds was in agreement with the previous report which also suggested that a marked increase in the fraction of root biomass might have assisted an increased N uptake (Singh et al., 2014). When a nutrient such as P is limiting, excess N in the tissue is often stored as the nitrogenous compounds such as amino acids or protein as observed in this study and elsewhere (Israel and Rufty, 1988; Staswick et al., 1991). In fact, there was a strong relationship (*r*^2^ = 0.88, *P* = 0.0005) between the measured protein concentrations and that estimated from seed N across CO_2_ (**Figure 5G**). Lower C/N or higher N/P ratios in response to P-deficiency have been observed previously and might be attributed to lower tissue P and carbon-rich components, such as oil, without a commensurate decrease in the N or N-rich compounds (Vasilas et al., 1984; Singh et al., 2014).

The eCO_2_-mediated decreases in leaf N concentration have been reported across P nutrition in soybean and cotton (Singh et al., 2013, 2014). In this study, eCO_2_ decreased seed N concentration, primarily under P-stress. The increase in C/N ratio at eCO_2_ in soybean seed was minor (1.3–3.2%) and consistent with the observation made in wheat grain (Högy et al., 2013). The seed N/P ratio also increased at eCO_2_, when averaged across P treatments, whereas it was not observed in soybean leaves (Singh et al., 2014).

The decrease in K concentration under P-deficiency was in agreement with a previous report and reflected an association between P nutrition status during plant growth and seed K concentration (Krueger et al., 2013). The current study indicated that soybean seed experiences lower Mg, S, Cu, B, and Zn concentrations in P-deficient soils, thus affecting its nutritional quality. The Zn is an essential mineral nutrient in the human diet and necessary for the proper function of immune system and to avoid stunted growth in infants and children (cf. Myers et al., 2015). The observed high Fe concentration under eCO_2_ contrasted the earlier reports of either lack of CO_2_ effect (Prior et al., 2008) or increased iron concentration at eCO_2_ in soybean (Myers et al., 2014). The nutrient status of the rooting medium during the course of experiment significantly affected the mineral uptake and their tissue concentrations (Conroy, 1992; Prior et al., 2008). A continuous supply of Fe and larger root growth at eCO_2_ might have facilitated enhanced Fe acquisition in this study resulting into the increased seed iron concentration (Wiersma, 2005).

Sucrose was the most abundant carbohydrate across P nutrition indicating its principal role in energy and carbon supply for the metabolic processes (Fait et al., 2006). The decrease in sucrose concentration under P-stress implied its utilization in the biosynthesis of other seed components. Sucrose might serve as an efficient medium for carbohydrate transport and as a precursor for biosynthetic pathways after hydrolysis into glucose and fructose. Diminished sucrose concentrations under P deficiency were accompanied by enhanced levels of glucose, ribose, and the sugar alcohols, myo-inositol, mannitol, and pinitol, that previously have been shown to accumulate in response to abiotic stresses and might serve as osmolytes to protect cellular structures during stress and the seed desiccation process (Fait et al., 2006; Barnaby et al., 2015). The eCO_2_ tended to decrease seeds carbohydrates as also reported previously in wheat grains (Högy et al., 2013).

The concentrations of organic acids including TCA intermediates have been studied in plant leaves, but responses of organic acids to P deficiency and eCO_2_ in seeds of major crops are limited (Fait et al., 2006; Barnaby et al., 2015; Sicher, 2015). Organic acids, particularly those that are TCA cycle intermediates, play a vital role in metabolic processes including biosynthesis of secondary metabolites, fatty acids and amino acids (Nelson and Cox, 2008). One of the plausible causes of the organic acid accumulation under P-stress might be its reduced utilization into fatty acid biosynthesis as shown by lower oil concentrations. However, a concurrent accumulation of succinate and amino acids were also reported previously in maturing seeds (Fait et al., 2006). Glycerate is involved in fatty acid metabolism, and its phosphate derivatives are key biochemical intermediates in the glycolytic pathway that converts glucose into pyruvate (Nelson and Cox, 2008). Decreased concentrations of many TCA cycle intermediate under eCO_2_ was in agreement with the previous study in soybean leaves (Sicher, 2015).

The consistent increase of free amino acids in P-deficient seeds implied their reduced utilization into the protein lattice as reported previously (Ranjan et al., 1962; Fait et al., 2006). Greater amino acid levels in P-deficient seeds might be attributed to depressed soybean growth rates and varying desiccation processes during the seed maturation. Fait et al. (2006) found an association between increased amino acid levels and mRNA encoding enzymes of amino acid biosynthesis indicating their accumulation during seed desiccation. Among measured amino acids, Gln was the most abundant amino acid in seeds, which was in agreement with the previous finding for other legumes (Yemane and Skjelvåg, 2003). The accumulation of Pro and Putrescine (a polyamine which might resutl form the breakdown of amino acids) in plant tissues in response to abiotic stress likely functions as an osmotic adjustment and as a defense against the reactive oxygen species (Bouchereau et al., 1999; Barnaby et al., 2015). Putrescine also plays a role in seed germination, and its external application has been reported to enhance pod retention and seed yield in Chickpea (*Cicer arietinum* L.) under cold stress (Nayyar, 2005). A general decrease in seed amino acids at eCO_2_ was in agreement with other C_3_ species (Högy et al., 2013). Interestingly, concentration of several amino acids showed P × CO_2_ interaction, which was attributable to their substantial reduction at eCO_2_ under P starvation, but not under control.

Regardless of CO_2_ treatment, the P starvation shifted the balance between protein and oil concentrations in soybean seeds. Similar changes exhibiting an inverse relationship between the seed protein and oil concentrations have also been reported previously under various environmental conditions (Dornbos and Mullen, 1992; Fenner, 1992; Rotundo and Westgate, 2009; Rotundo et al., 2009; Singh et al., 2012). The P deficiency appeared to show a trade-off between these two seed components by increasing (≈ 4.7%) the protein while decreasing (≈ 6.4%) the oil concentration by a comparable amount. During the seed filling processes, the accumulation of protein depends more on the remobilized resources from leaves as compared to the oil which mostly rely on the current photosynthesis (Rotundo and Westgate, 2009, 2010). The deceased photosynthesis under P deficiency in soybean has previously been reported as a part of this study (Singh et al., 2014; Singh and Reddy, 2016) and by others (Cure et al., 1988; Israel and Rufty, 1988) which might have led to the lower seed oil concentration. However, the eCO_2_ enhanced seed oil concentration (1.7–2.5%) due to a direct stimulatory effect on the photosynthetic processes that was observed in the previous reports (Singh et al., 2014; Singh and Reddy, 2016). Similar to the observation made in this study, Myers et al. (2014) did not find a significant effect of CO_2_ on the protein concentration in soybean seeds. However, total seed protein and oil yields were greater at eCO_2_ due to increase in seed productivity in this study.

Lower concentration of mineral elements, soluble sugars, and N-rich compounds such as protein and amino acids in plant tissues including seeds under eCO_2_ have been frequently reported and found to vary among cultivars or species (Prior et al., 1998, 2003; Gifford et al., 2000; Högy et al., 2013; Myers et al., 2014). Increased plant size and accumulation of photosynthates in plant tissues might partly explain the reduction of mineral concentrations and other components at eCO_2_ due to ‘carbohydrate dilution’ (Gifford et al., 2000; Prior et al., 2008). However, concentrations of not all constituents were decreased in response to eCO_2_ suggesting possibilities of other mechanisms such as lower transpiration-driven mass flow of nutrients, alteration in the uptake and assimilation, and an increased leaf thickness might also contribute to the lower mineral concentration at eCO_2_ in plant tissues (Taub and Wang, 2008; Myers et al., 2014 and references therein). In contrast to the concentration, the total yield (i.e., content) of a majority of the seed components was increased in response to eCO_2_, which was closely associated with the increases in the seed production. For example, the degree of eCO_2_-mediated enhancement in the yield of various seed components systematically declined as the P supply was reduced, which resulted in a minimal response of eCO_2_ at the lowest P treatment. This also resulted in to the observed P × CO_2_ interaction for yields of several seed components. The minimal response was attributable to the lack of stimulatory effects of eCO_2_ on seed production and various growth and physiological traits under severe P deficiency as found in previous reports (Singh et al., 2014, 2015). Since components yield is a function of the concentration of a given component and total seed production, factors affecting the plant growth and production will also influence the yields of seed components on the per plant or area basis.

In summary, this study largely supported the first hypothesis that P starvation will alter the concentration, but decrease the yield of seed components. P treatment significantly altered the concentration of 36 out of 38 seed components, of which 25 showed increasing while 11 showed decreasing patterns under P deficiency. Thus, P deficiency will alter the concentration seed components likely by reducing oil and mineral elements, but enhancing majority of other components including protein and the stress-responsive metabolites such as sugar alcohols, proline, and glycerate in soybean seeds across CO_2_ levels. The P treatment also affected the yield of 36 seed components and on an average majority of them declined except amino acids under P-stress. Remarkably, a number of amino acids compensated, at least partially, the drop in their total yield, especially due to several-fold enhancements in their concentrations under P-stress. Results also partly supported the second hypothesis that eCO_2_ will decrease the concentration but increase the yields of seed components which will depend on the P nutritional-status of soybean. The CO_2_ treatment significantly affected the concentrations of 28 out of 38 seed components, of which 23 (e.g., P, K, B, sucrose, glucose, citrate, aconitate, and several amino acids) showed decreasing while other (C, iron, Mn, Glycerate, and oil) components showed increasing patterns at eCO_2_. Furthermore, CO_2_ significantly affected the yield of 27 seed components and majority of them increased, especially at control and 0.10 mM P nutrition. In addition, the irresponsiveness of several amino acids concentration and yields of majority of components to eCO_2_ under control and the lowest P treatment, respectively, resulted into P × CO_2_ interaction. Thus, eCO_2_ appears to compensate, at least partially, for the overall yield of several seed components due to increased seed production, particularly near sufficient P nutrition.

## Author Contributions

SS and VR conceived and designed the experiment. SS conducted the experiment, collected and analyzed the data, and wrote the manuscript. JB and RS contributed in the seed metabolites analysis and assisted with the manuscript preparation. All author approved the final manuscript.

## Conflict of Interest Statement

The authors declare that the research was conducted in the absence of any commercial or financial relationships that could be construed as a potential conflict of interest.
